# Analysis of Severe Illness After Postvaccination COVID-19 Breakthrough Among Adults With and Without HIV in the US

**DOI:** 10.1001/jamanetworkopen.2022.36397

**Published:** 2022-10-13

**Authors:** Raynell Lang, Elizabeth Humes, Sally B. Coburn, Michael A. Horberg, Lily F. Fathi, Eric Watson, Celeena R. Jefferson, Lesley S. Park, Kirsha S. Gordon, Kathleen M. Akgün, Amy C. Justice, Sonia Napravnik, Jessie K. Edwards, Lindsay E. Browne, Deana M. Agil, Michael J. Silverberg, Jacek Skarbinski, Wendy A. Leyden, Cameron Stewart, Brenna C. Hogan, Kelly A. Gebo, Vincent C. Marconi, Carolyn F. Williams, Keri N. Althoff

**Affiliations:** 1 Department of Epidemiology, Johns Hopkins Bloomberg School of Public Health, Baltimore, Maryland; 2 Department of Medicine, University of Calgary, Calgary, Canada; 3 Kaiser Permanente Mid-Atlantic States, Mid-Atlantic Permanente Research Institute, Rockville, Maryland; 4 Stanford Center for Population Health Sciences, Palo Alto, California; 5 VA Connecticut Healthcare System, West Haven; 6 Department of Health Policy and Management, Yale School of Public Health, New Haven, Connecticut; 7 Department of Medicine, Yale School of Medicine, New Haven, Connecticut; 8 Department of Epidemiology, University of North Carolina at Chapel Hill, Chapel Hill; 9 Department of Medicine, School of Medicine, University of North Carolina at Chapel Hill, Chapel Hill; 10 Division of Research, Kaiser Permanente Northern California, Oakland; 11 Department of Infectious Diseases, Oakland Medical Center, Oakland, California; 12 Department of Medicine, Johns Hopkins School of Medicine, Baltimore, Maryland; 13 Emory University School of Medicine, Atlanta, Georgia; 14 Rollins School of Public Health, Atlanta, Georgia; 15 Atlanta Veterans Affairs Medical Center, Decatur, Georgia; 16 Epidemiology Branch, Division of AIDS at National Institute of Allergy and Infectious Diseases, National Institute of Health, Rockville, Maryland

## Abstract

**Importance:**

Understanding the severity of postvaccination SARS-CoV-2 (ie, COVID-19) breakthrough illness among people with HIV (PWH) can inform vaccine guidelines and risk-reduction recommendations.

**Objective:**

To estimate the rate and risk of severe breakthrough illness among vaccinated PWH and people without HIV (PWoH) who experience a breakthrough infection.

**Design, Setting, and Participants:**

In this cohort study, the Corona-Infectious-Virus Epidemiology Team (CIVET-II) collaboration included adults (aged ≥18 years) with HIV who were receiving care and were fully vaccinated by June 30, 2021, along with PWoH matched according to date fully vaccinated, age group, race, ethnicity, and sex from 4 US integrated health systems and academic centers. Those with postvaccination COVID-19 breakthrough before December 31, 2021, were eligible.

**Exposures:**

HIV infection.

**Main Outcomes and Measures:**

The main outcome was severe COVID-19 breakthrough illness, defined as hospitalization within 28 days after a breakthrough SARS-CoV-2 infection with a primary or secondary COVID-19 discharge diagnosis. Discrete time proportional hazards models estimated adjusted hazard ratios (aHRs) and 95% CIs of severe breakthrough illness within 28 days of breakthrough COVID-19 by HIV status adjusting for demographic variables, COVID-19 vaccine type, and clinical factors. The proportion of patients who received mechanical ventilation or died was compared by HIV status.

**Results:**

Among 3649 patients with breakthrough COVID-19 (1241 PWH and 2408 PWoH), most were aged 55 years or older (2182 patients [59.8%]) and male (3244 patients [88.9%]). The cumulative incidence of severe illness in the first 28 days was low and comparable between PWoH and PWH (7.3% vs 6.7%; risk difference, −0.67%; 95% CI, −2.58% to 1.23%). The risk of severe breakthrough illness was 59% higher in PWH with CD4 cell counts less than 350 cells/μL compared with PWoH (aHR, 1.59; 95% CI, 0.99 to 2.46; *P* = .049). In multivariable analyses among PWH, being female, older, having a cancer diagnosis, and lower CD4 cell count were associated with increased risk of severe breakthrough illness, whereas previous COVID-19 was associated with reduced risk. Among 249 hospitalized patients, 24 (9.6%) were mechanically ventilated and 20 (8.0%) died, with no difference by HIV status.

**Conclusions and Relevance:**

In this cohort study, the risk of severe COVID-19 breakthrough illness within 28 days of a breakthrough infection was low among vaccinated PWH and PWoH. PWH with moderate or severe immune suppression had a higher risk of severe breakthrough infection and should be included in groups prioritized for additional vaccine doses and risk-reduction strategies.

## Key Points

**Question:**

In 2021, among fully vaccinated people with breakthrough COVID-19 illness, was the risk of severe illness higher for people with HIV (PWH) compared with people without HIV (PWoH)?

**Findings:**

In this cohort study of 3649 patients with breakthrough COVID-19, there was no overall difference in risk of severe disease between PWH and PWoH. PWH with CD4 cell count less than 350 cells/μL had a 59% increased risk of severe breakthrough illness compared with PWoH.

**Meaning:**

Although vaccinations effectively reduce the risk of severe COVID-19 illness in both PWH and PWoH, these findings suggest that PWH with moderate or severe immune suppression (CD4 cell count <350 cells/μL) could be at higher risk of severe breakthrough infection compared with PWoH, and PWH with moderate immune suppression should be considered for additional vaccine dosages and other risk-reduction measures.

## Teaser

This cohort study estimates the rate and risk of severe breakthrough COVID-19 illness among vaccinated people with HIV and people without HIV who experience a breakthrough infection.

## Introduction

Vaccination for SARS-CoV-2 is an effective protective measure against COVID-19.^1,2,3^ Observational studies^4,5,6,7,8,9^ suggest that people with HIV (PWH) have a higher risk for postvaccination SARS-CoV-2 infection (ie, breakthrough) than people without HIV (PWoH). Data on breakthrough COVID-19 illness, particularly severe illness requiring hospitalization, for PWH remain sparse.^10,11^

Differences in COVID-19 illness severity by HIV status are unknown, with some studies finding comparable severity^12,13,14,15^ and others reporting increased severity among PWH compared with PWoH.^16,17,18,19,20^ Limitations of prior studies included sample size, lack of comparator populations of PWoH, residual confounding in observational studies, and occurrence before COVID-19 vaccine availability. Large-scale data are needed to observe a sufficient sample size of breakthrough illness for addressing risk factors associated with severe illness, by HIV status and immune suppression among PWH.

Current US Centers for Disease Control and Prevention (CDC) guidelines recommend risk reduction behaviors (ie, mask wearing), an additional COVID-19 primary series vaccine dose, and a second booster dose for PWH with “advanced or untreated HIV infection.”^21,22^ PWH with partially recovered CD4 cell counts (>200 cells/μL) and moderate immune suppression are not currently recommended for an additional or second booster dose. Our objective was to determine whether HIV infection was associated with increased severe COVID-19 illness among fully vaccinated adults with a breakthrough SARS-CoV-2 infection and to identify the factors associated with severe COVID-19 breakthrough illness among PWH, including level of immune suppression and HIV viral replication.

## Methods

### Study Population

The Corona-Infectious-Virus Epidemiology Team (CIVET-II) cohort is composed of 4 cohorts including Kaiser Permanente Mid-Atlantic States (Maryland, District of Columbia, and northern Virginia), Kaiser Permanente Northern California, University of North Carolina Chapel Hill HIV Clinic, and the Veterans Aging Cohort Study (VACS), a cohort of PWH (and similar PWoH) receiving care within the National US Veterans Affairs Healthcare System. This collaboration is an extension of the North American AIDS Cohort Collaboration on Research and Design.^23^ Cohorts received approval (including waivers and/or exemptions of consent when necessary) from their local institutional review boards. Approval for this cohort study was obtained from the Johns Hopkins Bloomberg School of Public Health institutional review board. Strengthening the Reporting of Observational Studies in Epidemiology (STROBE) reporting guidelines were followed.

Adults (aged ≥18 years) who were receiving care at 1 of the 4 institutions (eTable 1 in the Supplement) and were fully vaccinated against COVID-19 between December 11, 2020 (Emergency Use Authorization of the first COVID-19 vaccine), and June 30, 2021, were identified. Full vaccination status was defined using CDC criteria: (1) 14 days after BNT162 (Pfizer) or mRNA-1273 (Moderna) mRNA vaccine second dose or (2) 14 days after Janssen Ad26.COV2.S (Johnson & Johnson) single dose.^24^ Three PWoH were matched to each PWH according to the date fully vaccinated (within 14 days of the PWH vaccination date), 10-year age group, race, ethnicity, and sex at birth within each contributing cohort. Race and ethnicity were extracted from electronic health records (EHRs) with both self-report and clinician-observed sources. Race and ethnicity were included because of the disproportionate burdens of HIV by race and ethnicity. The VACS (67 627 patients) matches each veteran with HIV to 2 veterans without HIV according to age, race, ethnicity, sex, and clinical site at cohort entry; VACS participants were not matched by date fully vaccinated.^25^

The study population for this nested study included all those with observed COVID-19 breakthrough, defined as the first detected SARS-CoV-2 infection (detectable SARS-CoV-2 nucleic acid amplification assay or antigen test) or COVID-19 diagnosis using *International Statistical Classification of Diseases and Related Health Problems, Tenth Revision (ICD-10)* codes (eTable 2 in the Supplement) after the date fully vaccinated. If a COVID-19 diagnosis code was also identified within the 90-day window of detectable result, the first laboratory test was the diagnosis date. All variables were abstracted from EHRs.

### Outcome: Severe COVID-19 Breakthrough Illness

Severe COVID-19 breakthrough illness was defined as (1) hospitalization within 28 days of breakthrough and (2) discharge diagnosis ranked first or second was COVID-19. All discharge diagnoses were evaluated by 1 infectious disease physician (R.L.) to exclude hospitalizations that were unlikely to be due to COVID-19 (trauma, surgical, non–COVID-19 infections, mental health, or substance use admissions). Only the first-ranked discharge diagnosis was available for 1 cohort (contributing 28% of the study population). In this cohort, if COVID-19 was not the first-ranked discharge diagnosis and there was 1 or more other discharge diagnosis suggestive of COVID-19 (ie, acute respiratory failure), the patient was classified as having severe COVID-19 breakthrough.

Mechanical ventilation and extracorporeal membrane oxygenation procedures were extracted from *ICD-10* procedure and *Current Procedural Terminology* codes (eTable 2 in the Supplement). Death occurring during or within 30 days following hospitalization or within 30 days following COVID-19 diagnosis among those not hospitalized was extracted from EHRs.

### Exposure: HIV Infection

PWH were identified using HIV registries or HIV *ICD-10* diagnosis codes (eTable 1 in the Supplement). PWoH had no evidence of HIV infection using these same sources as of December 11, 2020.

### Covariates

Demographic covariates included age, race, ethnicity, and birth sex. COVID-19 covariates included the primary series vaccine type (Pfizer, Moderna, Johnson & Johnson), additional vaccine dose (receipt ≥28 days after completion of primary series), and SARS-CoV-2 infection before the date fully vaccinated (history of COVID-19).

Comorbidity covariates included obesity (body mass index [calculated as weight in kilograms divided by height in meters squared] ≥30), type 2 diabetes, hypertension, end-stage kidney disease (ESKD), immune suppressive conditions (tissue or solid organ transplantation [SOT], rheumatoid arthritis [RA], systemic lupus erythematosus [SLE]), cancer, and pregnancy. Comorbidity diagnoses were identified using *ICD-10* codes, measured closest to the date fully vaccinated and after October 1, 2015, or January 1, 2020, for cancer and pregnancy only (eTable 2 in the Supplement).

Among PWH, CD4 cell count and HIV-1 plasma RNA viral load suppression were collected closest to the date fully vaccinated after January 1, 2020, and at antiretroviral therapy initiation (from 12 months before to 1 month after). HIV viral suppression was defined as less than 50 copies/mL. History of AIDS diagnosis (clinical diagnosis^26^ or CD4 cell count <200 cells/μL) before date fully vaccinated was included.

### Statistical Analysis

Study entry was the date of observed breakthrough COVID-19. Individuals were followed to the first date of severe breakthrough COVID-19 illness (outcome) or date of death, disenrollment from the health system, 28 days after breakthrough COVID-19, or December 31, 2021.

Severe COVID-19 breakthrough illness monthly incidence rates (IRs) per 100 person-years and 95% CIs were calculated by HIV status. Severe COVID-19 breakthrough cumulative incidence was estimated from the date of breakthrough through day 28; estimates were stratified by HIV status, and among PWH, CD4 cell count, and viral suppression. Using the same timescale, cumulative incidence was estimated by HIV status for each vaccine type and for those who received an additional vaccine dose (≥28 days after primary series completion and before breakthrough COVID-19). Log-rank tests were calculated to test for differences in cumulative incidence, and risk differences were estimated with SE and Wald 95% CI.

A discrete time-to-event analysis using a complimentary log-log model estimated the unadjusted and adjusted hazard ratios (aHRs) with 95% CI for severe COVID-19 breakthrough illness risk by HIV status. Adjustment factors included sex, race, ethnicity, age, additional vaccine dose following primary series, prior COVID-19, obesity, diabetes, hypertension, ESKD, SOT, RA, SLE, cancer, and cohort. Among PWH, prior AIDS diagnosis, HIV viral suppression, and CD4 cell count were evaluated as risk factors for severe COVID-19 in a subgroup analysis. Sensitivity analyses excluded participants without first or second diagnosis code rankings. Analyses were conducted with R statistical software version 4.1.2 (R Project for Statistical Computing). Two-sided *P* < .05 was considered significant.

## Results

Among 113 994 patients (33 029 PWH and 80 965 PWoH), 3649 experienced breakthrough COVID-19 (1241 PWH and 2408 PWoH); 2182 patients (59.8%) were aged 55 years or older, 3244 patients (88.9%) were male, and 1706 (46.8%) were non-Hispanic Black. Among those with breakthrough COVID-19, 2158 (59.1%) received Pfizer and 1140 (31.2%) received Moderna for their primary series; 546 patients (15.0%) received an additional COVID-19 vaccine dose 28 days or more after primary series completion (242 PWH [19.5%] and 304 PWoH [12.6%]). The median (IQR) time from primary series completion to additional dose was 188 (151-220) days among PWH and 221 (200-242) days among PWoH (eFigure 1 in the Supplement). The majority of observed COVID-19 breakthroughs were laboratory confirmed (2740 breakthroughs [75.1%]); 1771 breakthroughs (48.5%) occurred during the Delta variant (B.1.617.2) surge from July to October 2021, and 1497 (41.0%) occurred from November to December 2021 (Omicron variant B.1.1.529 wave) (Table 1). Among PWH, 325 (26.2%) had a history of AIDS, 1016 (90.6%) were virally suppressed, and the median (IQR) CD4 cell count was 620 (438-846) cells/μL at the time fully vaccinated. A lower proportion of PWH than PWoH had obesity, diabetes, hypertension, or ESKD at the date fully vaccinated (829 patients [66.8%] vs 1865 patients [77.5%]). PWH with a breakthrough COVID-19 illness who had a CD4 cell count of less than 200 cells/μL (9 of 54 patients [16.7%]) and 200 to 349 cells/μL(17 of 109 patients [15.6%]) were less likely to receive an additional vaccine dose, compared with PWH with CD4 cell counts greater than or equal to 350 cells/μL(182 of 899 patients [20.5%]) (eTable 3 in the Supplement).

**Table 1.** Characteristics of Patients at Date of SARS-CoV-2 Breakthrough Infection

Characteristic	Patients, No. (%) (N = 3649)	
	PWoH (n = 2408)	PWH (n = 1241)
Hospitalization	170 (7.1)	79 (6.4)
Mechanical ventilation among those hospitalized^a^	16 (9.4)	8 (10.1)
Deaths among breakthrough infections^b^	21 (0.9)	12 (1.0)
Age, y^c^		
18-24	9 (0.4)	9 (1.7)
25-34	137 (5.7)	112 (9.0)
35-44	279 (11.6)	184 (14.8)
45-54	492 (20.4)	245 (19.7)
55-64	797 (33.1)	359 (28.9)
65-74	539 (22.4)	264 (21.3)
≥75	155 (6.4)	68 (5.5)
Sex		
Male	2108 (87.5)	1136 (91.5)
Female	300 (12.5)	105 (8.5)
Ethnicity and race		
Hispanic	366 (15.2)	183 (14.7)
Non-Hispanic Black or African American	1151 (47.8)	555 (44.7)
Non-Hispanic White	744 (30.9)	421 (33.9)
Other^d^	131 (5.4)	71 (5.7)
Unknown	16 (0.7)	11 (0.9)
Month of breakthrough		
January-June 2021	247 (10.3)	134 (10.8)
July-October 2021	1132 (47.0)	639 (51.5)
November-December 2021	1029 (42.7)	468 (37.7)
Primary vaccination series type		
Pfizer	1194 (49.6)	605 (48.8)
Pfizer plus third dose	194 (8.1)	165 (13.3)
Moderna	676 (28.1)	301 (24.3)
Moderna plus third dose	95 (3.9)	68 (5.5)
Johnson & Johnson	234 (9.7)	93 (7.5)
Johnson & Johnson plus second dose	15 (0.6)	9 (0.7)
COVID-19 before full vaccination	292 (12.1)	176 (14.2)
Comorbidities at full vaccination^e^		
Obese body mass index (≥30)^f^	1218 (53.6)	434 (36.1)
Diabetes	817 (33.9)	308 (24.8)
Hypertension	1443 (59.9)	633 (51.0)
End-stage kidney disease	63 (2.6)	38 (3.1)
Immune suppressive conditions at full vaccination		
Organ or tissue transplantation^e^	43 (1.8)	18 (1.5)
Rheumatoid arthritis^e^	44 (1.8)	14 (1.1)
Systemic lupus erythematosus^e^	10 (0.4)	3 (0.2)
Cancer diagnosis^g^	187 (7.8)	129 (10.4)
Pregnancy^g^	4 (0.2)	3 (0.2)
CD4 cell count at antiretroviral therapy initiation, median (IQR), cells/μL	NA	393 (234-613)
Unknown	NA	536 (43.2)
AIDS before full vaccination^h^	NA	325 (26.2)
CD4 cell count at full vaccination		
Median (IQR), cells/μL	NA	620 (438-846)
<200	NA	54 (4.4)
200-349	NA	109 (8.8)
350-499	NA	182 (14.6)
≥500	NA	707 (57.0)
Unknown	NA	189 (15.2)
Suppressed HIV RNA at full vaccination (<50 copies/mL)	NA	1016 (90.6)
Unknown	NA	120 (9.7)

Abbreviations: PWH, people with HIV; PWoH, people without HIV.

^a^ Mechanical ventilation was measured during the dates of hospital admission and discharge.

^b^ Death was defined as dying while hospitalized for COVID-19 or within 30 days following discharge or within 30 days of COVID-19 diagnosis among those not hospitalized.

^c^ Age was categorized in 5-year increments for descriptive purposes.

^d^ Other race includes Asian, Native Hawaiian or other Pacific Islander, American Indian or Alaska Native, and 1 or more race.

^e^ Body mass index, diabetes, hypertension, end-stage kidney disease, organ or tissue transplantation, rheumatoid arthritis, and systemic lupus erythematosus were measured as close to the date fully vaccinated as possible within the window of October 1, 2015, to the date fully vaccinated.

^f^ Body mass index is calculated as weight in kilograms divided by height in meters squared.

^g^ Cancer and pregnancy were measured as close to the date fully vaccinated as possible within the window of January 1, 2020, to the date fully vaccinated.

^h^ AIDS was defined using clinical diagnosis codes of AIDS-defining conditions.

Two hundred forty-nine participants (6.8% overall; 79 PWH [6.4%]; 170 PWoH [7.1%]) had severe COVID-19 breakthrough illness and were hospitalized (Table 1 and eFigure 2 in the Supplement). Most hospitalizations (163 hospitalizations [65.5%]) occurred on the same day, and 202 hospitalizations (81.1%) occurred within 2 days following COVID-19 diagnosis. The greater proportion of those with obesity, diabetes, hypertension, or ESKD at the date fully vaccinated in PWoH compared with PWH persisted among those who were hospitalized (160 patients [94.1%] vs 65 patients [82.3%]). The median (IQR) duration of hospitalization was 5 (3-8) days among PWoH and 4 (2-8) days among PWH. Among all hospitalized patients, 24 (9.6%) were mechanically ventilated (Table 1). There were 33 deaths (0.9% overall; 12 PWH [1.0%]; 21 PWoH [0.8%]) among all those with COVID-19 breakthrough illness that occurred within 30 days of COVID-19 diagnosis. Among the 249 patients hospitalized, there were 20 deaths (8.0%) that occurred while the patients were hospitalized or within 30 days of discharge. eTable 4 in the Supplement describes characteristics stratified by HIV and severe COVID-19 breakthrough illness.

### IRs and Cumulative Incidence of Severe COVID-19 Breakthrough Illness

**Figure 1.**
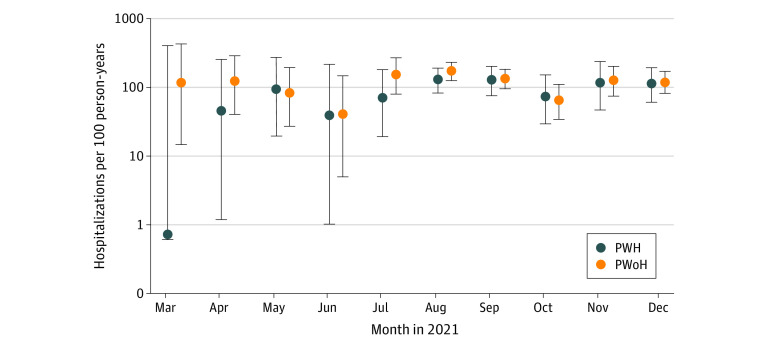
Incidence Rates of Severe COVID-19 Breakthrough per 100 Patient-Years in People With HIV (PWH) and People Without HIV (PWoH) by Month Data for 3649 patients are shown. The incidence rate estimates for January 2021 were not estimated as there were 0.00 and 0.02 person-years of observation after COVID-19 breakthrough infection in PWH and PWoH, respectively. Similarly, the person-years in February 2021 were 0.14 and 0.21 in PWH and PWoH, respectively, and there were no severe COVID-19 breakthrough illness events; incidence rates were not estimated. Error bars denote 95% CIs.

The IRs of severe COVID-19 breakthrough illness were similar among PWoH (138 cases per 100 person-years; 95% CI, 118-160 cases per 100 person-years) vs PWH (117 cases per 100 person-years; 95% CI, 92-145 cases per 100 person-years) and were stable over time (Figure 1 and eTable 5 in the Supplement). There were slight fluctuations reflecting the bimodal distribution of the Delta and Omicron variant waves (eFigure 3 in the Supplement).

**Figure 2.**
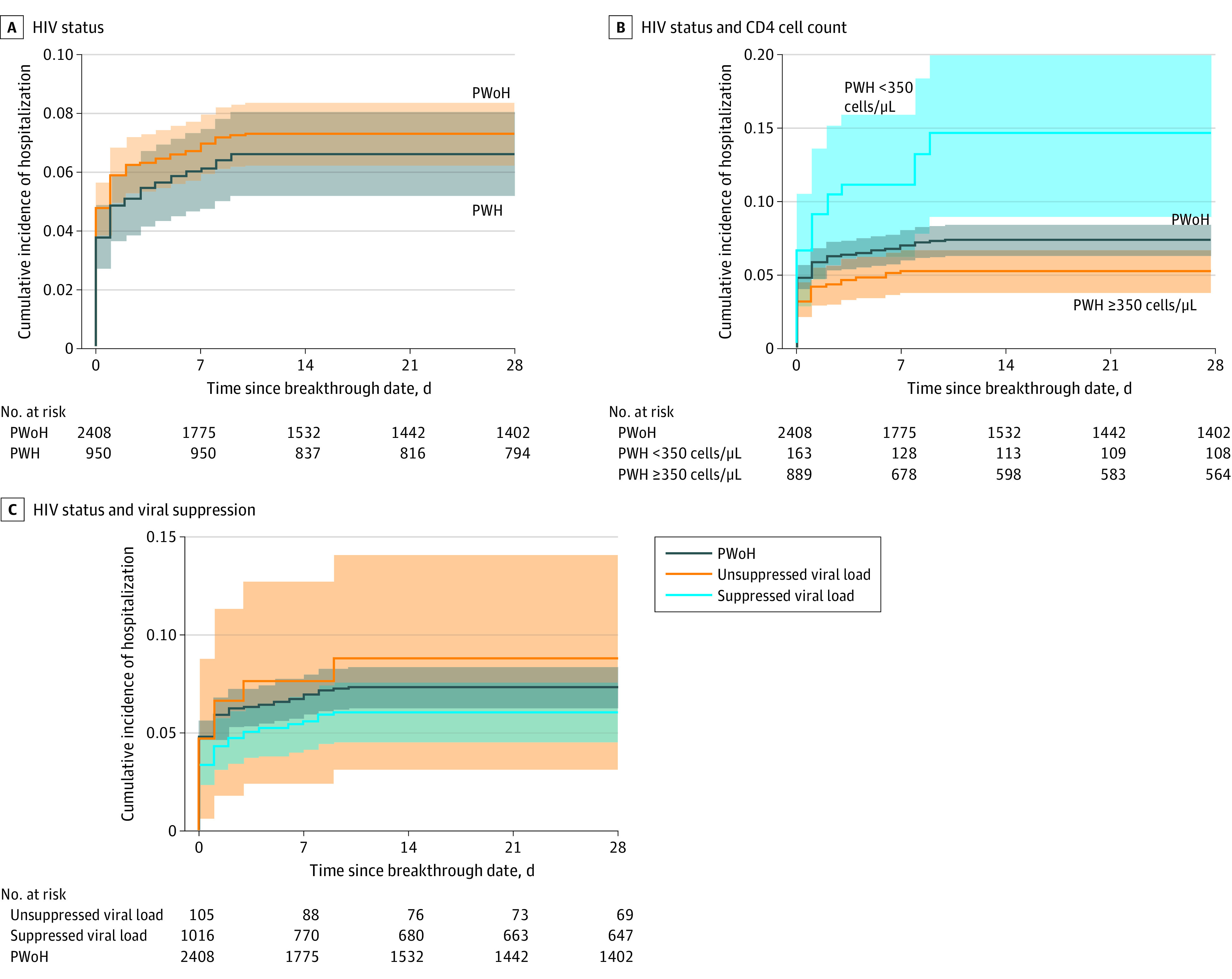
Cumulative Incidence of Severe COVID-19 Breakthrough Illness Among People With HIV (PWH) and People Without HIV (PWoH) Graphs show incidence of severe COVID-19 breakthrough illness by HIV status (A) (log-rank test *P* = .40), by CD4 cell count and HIV status (B) (log-rank test including PWoH, *P* < .001; log-rank test after excluding PWoH, *P* < .001), and by HIV viral suppression (defined as HIV-1 RNA viral load <50 copies/mL) and HIV status (C) (log-rank test including PWoH, *P* = .31; log-rank test after excluding PWoH, *P* = .28). Shaded areas denote 95% CIs.

The 28-day cumulative incidence of severe COVID-19 breakthrough illness was similar among PWoH vs PWH (7.3% [95% CI, 6.3% to 8.4%] vs 6.7% [95% CI, 5.2% to 8.1%]; log-rank *P* = .39; risk difference, –0.67% [95% CI, –2.58% to 1.23%]) (Figure 2A). PWH with lower CD4 cell counts (<350 cells/μL) at full vaccination had a higher risk of severe COVID-19 breakthrough illness compared with PWH with CD4 cell counts greater than or equal to 350 cells/μL and PWoH (Figure 2B and eFigure 4 in the Supplement). Risk did not differ by HIV viral load (Figure 2C).

**Figure 3.**
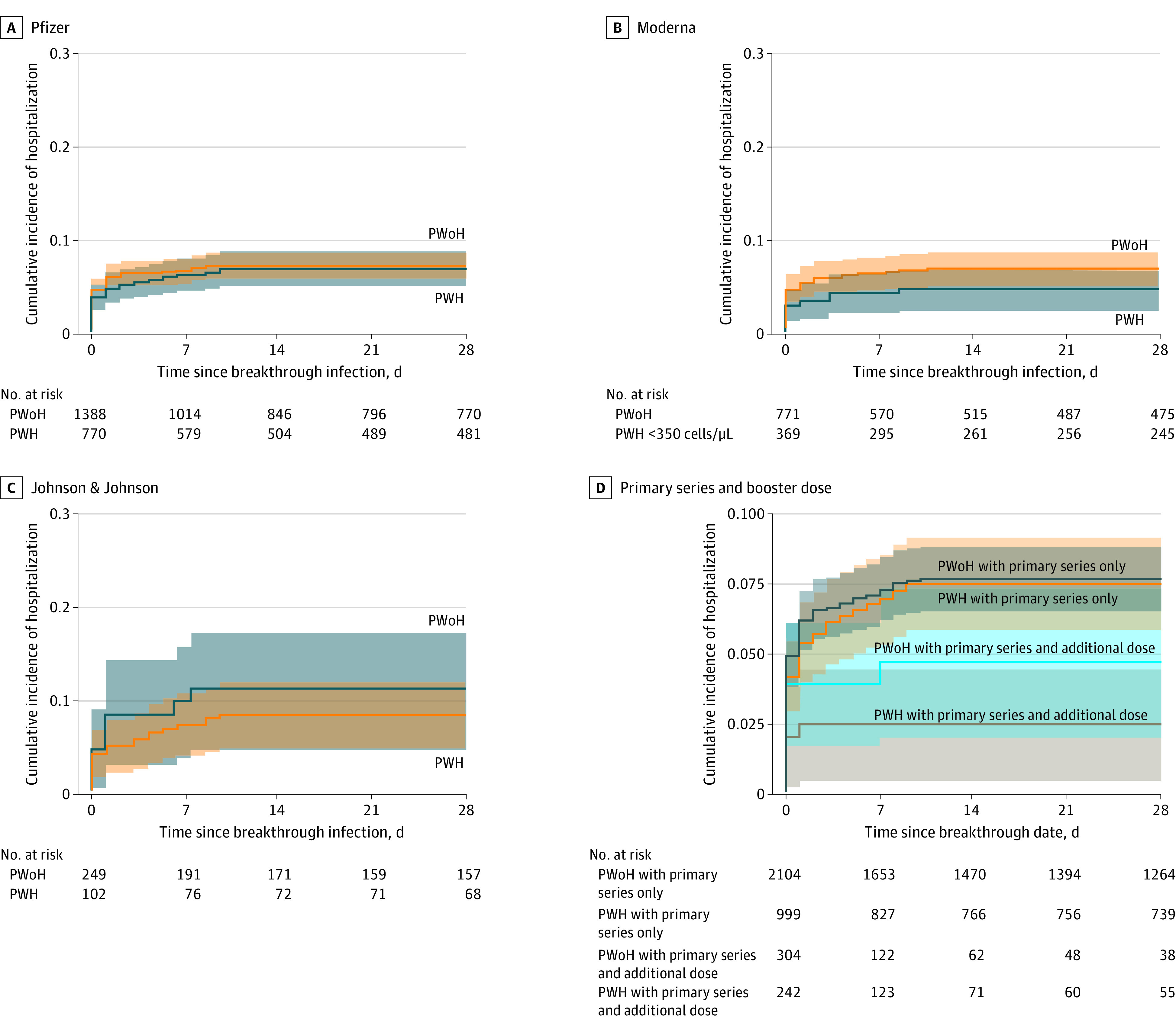
Cumulative Incidence of Severe COVID-19 Breakthrough Illness by HIV Status and Primary Vaccination Series Type Among People With HIV (PWH) and People Without HIV (PWoH) Graphs show incidence of illness by primary vaccination series type (A, log-rank test for Johnson & Johnson, *P* = .40; B, log-rank test for Pfizer, *P* = .67; C, log-rank test for Moderna, *P* = .15) and by primary series and additional vaccine doses (D, log-rank test, *P* = .02).Shaded areas denote 95% CIs.

Severe COVID-19 breakthrough illness risk was highest among patients with Johnson & Johnson primary vaccine series (9.3%; 95% CI, 6.1%-12.4%), followed by Pfizer (7.2%; 95% CI, 6.1%-8.3%), and Moderna (6.2%; 95% CI, 4.8%-7.7%) (Figure 3A), with no significant differences by HIV status within each vaccine group. Regardless of the primary vaccine series type, having an additional dose was associated with reduced risk of severe COVID-19 breakthrough illness in both PWH and PWoH (Figure 3B)

### Factors Associated With Risk of Severe COVID-19 Breakthrough Illness, by HIV Status

In unadjusted models, PWH with CD4 cell count less than 350 cells/μL had a higher risk of severe infection than PWoH (HR, 2.00; 95% CI, 1.26-3.02). In adjusted analyses, there was no difference in severe COVID-19 breakthrough illness risk among PWH vs PWoH (aHR, 1.02; 95% CI, 0.76-1.35). PWH with a CD4 cell count less than 350 cells/μL had a 59% increased risk of severe breakthrough illness compared with PWoH (aHR, 1.59; 95% CI, 0.99-2.46; *P* = .049) (Table 2). Stratified by HIV status, PWH and PWoH had an increased risk of severe COVID-19 breakthrough illness with increasing age and history of COVID-19 (before being fully vaccinated), and decreased risk among those with an additional vaccine dose.

**Table 2.** Crude HRs and aHRs and 95% CIs of Severe SARS-CoV-2 Breakthrough Illness

Characteristics	PWoH^a^		PWH^b^	
	HR (95% CI) (N = 2350)	aHR (95% CI) (n = 170)	HR (95% CI) (N = 1035)	aHR (95% CI) (n = 68)
Total population by HIV status (N = 3385)^c^				
PWoH	1 [Reference]	1 [Reference]	NA	NA
PWH, CD4 cell count <350 cells/μL	NA	NA	2.00 (1.26-3.02)	1.59 (0.99-2.46)
PWH, CD4 cell count ≥350 cells/μL	NA	NA	0.70 (0.50-0.97)	0.95 (0.67-1.32)
Sex				
Male	1 [Reference]	1 [Reference]	1 [Reference]	1 [Reference]
Female	0.25 (0.10-0.52)	0.48 (0.16-1.12)	1.50 (0.66-2.95)	2.88 (1.03-7.11)
Ethnicity and race				
Hispanic	1.01 (0.61-1.62)	1.21 (0.72-1.98)	0.98 (0.43-2.10)	1.54 (0.65-3.38)
Non-Hispanic Black or African American	1.24 (0.88-1.77)	1.26 (0.86-1.85)	1.26 (0.73-2.22)	1.26 (0.70-2.32)
Non-Hispanic White	1 [Reference]	1 [Reference]	1 [Reference]	1 [Reference]
Other or unknown^d^	0.52 (0.18-1.19)	1.24 (0.43-2.86)	1.30 (0.43-3.21)	2.71 (0.87-7.12)
Age, y				
<55	1 [Reference]	1 [Reference]	1 [Reference]	1 [Reference]
55-64	6.63 (3.54-13.80)	3.49 (1.74-7.86)	3.02 (1.52-6.33)	2.11 (0.98-4.80)
65-74	12.60 (6.82-26.00)	5.37 (2.64-12.20)	4.41 (2.25-9.13)	3.01 (1.37-6.98)
≥75	21.00 (10.70-45.00)	8.44 (3.90-20.00)	7.71 (3.25-17.9)	5.21 (1.88-14.40)
Primary vaccination series type				
Primary series only	1 [Reference]	1 [Reference]	1 [Reference]	1 [Reference]
Primary series plus additional dose	0.63 (0.34-1.07)	0.56 (0.29-0.98)	0.43 (0.17-0.91)	0.47 (0.18-1.04)
COVID-19 before full vaccination	0.39 (0.18-0.71)	0.21 (0.09-0.40)	0.43 (0.15-0.97)	0.22 (0.07-0.52)
Calendar period of breakthrough				
January-June 2021	1 [Reference]	NA	1 [Reference]	NA
July-October 2021	1.49 (0.91-2.62)	NA	2.53 (1.03-8.40)	NA
November-December 2021	0.77 (0.45-1.40)	NA	1.41 (0.53-4.89)	NA
Comorbidities before full vaccination				
Obese body mass index (≥30 vs <30)^e^	0.98 (0.72-1.34)	1.01 (0.73-1.40)	1.38 (0.85-2.23)	1.42 (0.83-2.43)
Diabetes	2.80 (2.07-3.81)	1.11 (0.79-1.56)	2.26 (1.39-3.64)	1.52 (0.88-2.60)
Hypertension	7.73 (4.65-14.00)	4.51 (2.35-9.80)	3.02 (1.78-5.39)	1.25 (0.66-2.47)
End-stage kidney disease	6.25 (3.88-9.58)	2.53 (1.33-4.56)	1.82 (0.55-4.41)	1.12 (0.31-3.09)
Immune suppressive conditions at full vaccination				
Organ or tissue transplantation	6.79 (3.89-11.0)	2.49 (1.20-4.96)	0.95 (0.05-4.27)	0.55 (0.03-3.39)
Rheumatoid arthritis	2.03 (0.80-4.19)	1.33 (0.50-2.87)	1.28 (0.07-5.78)	1.09 (0.06-5.45)
Cancer diagnosis	2.64 (1.75-3.86)	1.47 (0.95-2.19)	2.92 (1.64-4.95)	1.97 (1.05-3.51)
Systemic lupus erythematosus^f^	2.03 (0.80-4.19)	1.33 (0.50-2.87)	NA	NA
AIDS diagnosis before full vaccination	NA	NA	1.83 (1.11-2.96)	1.22 (0.70-2.10)
HIV RNA at full vaccination	NA	NA	NA	NA
Suppressed (<50 copies/mL)	NA	NA	1 [Reference]	NA
Unsuppressed (≥50 copies/mL)	NA	NA	1.43 (0.66-2.74)	NA
CD4 cell count at full vaccination, cells/μL				
≥500	NA	NA	1 [Reference]	1 [Reference]
350-499	NA	NA	1.28 (0.62-2.44)	1.19 (0.56-2.35)
200-349	NA	NA	2.52 (1.28-4.66)	1.65 (0.80-3.21)
<200	NA	NA	3.98 (1.86-7.76)	2.57 (1.15-5.29)

Abbreviations: aHR, adjusted hazard ratio; HR, crude hazard ratio; PWH, people with HIV; PWoH, people without HIV.

^a^ Adjusted for age (categorized into a 4-level group to assess a dose-response relationship among older people [ie, aged ≥55 years] who are known to be at greater risk), sex, race and ethnicity, primary vaccination series type, COVID-19 before full vaccination, comorbidities before full vaccination, immune suppressive conditions at full vaccination, and cohort.

^b^ Adjusted for the covariates in the table and cohort; 184 PWH (15.1%) were excluded because of missing CD4 or HIV RNA measurements.

^c^ The total number of severe COVID-19 illness events was 238.

^d^ Other race includes Asian, Native Hawaiian or other Pacific Islander, American Indian or Alaska Native, and 1 or more race.

^e^ Body mass index is calculated as weight in kilograms divided by height in meters squared.

^f^ There were no severe COVID-19 breakthrough illnesses among PWH with systemic lupus erythematosus, and it was therefore excluded from the multivariable model.

Among PWH, severe COVID-19 breakthrough risk increased with decreasing CD4 cell count (compared with CD4 cell count ≥500 cells/μL) (Table 2). Female PWH had a nearly 3-fold increased severe COVID-19 breakthrough risk vs male PWH. Increased risk associated with non-Hispanic Black race and Hispanic ethnicity and comorbidities ranged from 12% to 52%. Having a cancer diagnosis was associated with nearly 2-fold increased risk of severe COVID-19 breakthrough illness.

Among PWoH, women had a reduced risk of severe COVID-19 breakthrough illness compared with men (aHR, 0.48; 95% CI, 0.16-1.12) although the difference was not significant (Table 2). There was no observed difference in risk by race, obesity, diabetes, RA, SLE or having a cancer diagnosis, but there was a 4.51-fold increased risk with hypertension and 2.53-fold increased risk with ESKD and 2.49-fold increased risk with SOT. Sensitivity analyses excluding patients without diagnosis code rankings did not qualitatively change findings.

### Mechanical Ventilation and Death in Severe COVID-19 Breakthrough Illness

Among the 249 hospitalized patients, a greater proportion of patients with CD4 cell counts less than 350 cells/μL required mechanical ventilation or died during hospitalization compared with PWH with higher CD4 counts and PWoH (eFigure 5 in the Supplement). No patients received extracorporeal membrane oxygenation.

Patients who needed mechanical ventilation (24 patients) or died (33 patients) were older (aged ≥55 years), male, non-Hispanic Black, had high proportions of comorbidities, and low uptake (10%-13%) of additional vaccine doses (eTable 6 in the Supplement). Of the 20 known deaths during or within 30 days following COVID-19 hospitalization, most occurred in patients with obese body mass index (13 patients [65.0%]), hypertension (19 patients [95.0%]), diabetes (13 patients [65.0%]), ESKD (6 patients [30.0%]), SOT (4 patients [20.0%]), RA (2 patients [10.0%]), or cancer (5 patients [25.0%]). Among 6 PWH who died during a severe COVID-19 breakthrough illness, 3 (50.0%) had a prior diagnosis of AIDS and their median (IQR) CD4 cell count at full vaccination was 352 (291-423) cells/μL.

## Discussion

Prior CIVETs collaboration analyses^4^ showed a 28% increase in breakthrough COVID-19 among PWH vs PWoH. The findings of this cohort study showed that the risk of severe illness (requiring hospitalization) after COVID-19 breakthrough was low (6.8% of 3649 vaccinated PWH and PWoH) and did not differ by HIV status overall. PWH with lower CD4 cell counts (<350 cells/μL), however, had higher risk of severe COVID-19 breakthrough illness compared with PWoH, suggesting a role of immune dysfunction in the increased risk. The lack of difference in severe COVID-19 breakthrough illness risk between PWoH and PWH with higher CD4 cell counts may be associated with engagement in medical care, different health care–seeking behaviors, and reduced comorbidities among the PWH included compared with PWoH. The increased risk of severe COVID-19 breakthrough illness for PWH with moderate immune suppression (ie, CD4 cell count 200-349 cells/μL)^27^ (1) suggests that the recommendation for additional primary series vaccination doses should be expanded to PWH with moderate immune suppression, (2) supports the current recommendation of a first booster, and (3) suggests counseling on risk-reduction strategies among those with moderate immune suppression.

Prior studies^28,29^ have also demonstrated that greater immune dysfunction is associated with increased severe COVID-19 illness risk in PWH. Both CD4^+^ and CD8^+^ T cells have important roles in the viral immune response and are positively correlated with the antibody response to SARS-CoV-2.^29,30^ CD4^+^ T-cell function is needed for effective vaccine responses.^27,31^ The increased risk of severe COVID-19 breakthrough among PWH with lower CD4 cell counts is likely multifactorial and requires further investigation.

Sex, age, comorbidities, additional vaccine doses, and prior COVID-19 infection are associated with the risk of severe COVID-19 breakthrough illness.^10,32,33,34^ Among both PWoH and PWH, increasing age was the most significant factor associated with severe COVID-19 breakthrough illness in our study. Female PWoH had reduced risk, which has been previously documented^35,36,37^; however, female PWH had increased risk of severe illness. It is known that male and female individuals have distinct immune system responses, with female individuals often demonstrating increased immune competence and less inflammatory immune responses, possibly contributing to their reduced risk of severe COVID-19 breakthrough illness; however, immune dysfunction with HIV may alter this effect.^35,38,39^ Additionally, among the female PWH, a high proportion had obesity, which likely influenced the increased risk of severe breakthrough illness and warrants further investigation.

Despite recommendations for additional COVID-19 vaccine doses being based in part on CD4 cell count,^22^ we identified that the proportion of PWH who received additional doses was low, varied little by CD4 cell count, and was likely associated with clinical decision-making and patient preference. Prior COVID-19 has been found to reduce the risk of subsequent COVID-19 illness, particularly following vaccination^40^; our findings furthered this demonstrating a reduction in severe breakthrough COVID-19 among people with COVID-19 prior to full vaccination.

Among PWoH, several comorbidities have been associated with increased severe COVID-19 breakthrough illness risk.^33,34,41^ Our findings suggest an increased risk associated with hypertension, ESKD, and SOT. Comorbidities were prevalent among those who experienced a severe COVID-19 breakthrough illness with most having a diagnosis of obesity, diabetes, hypertension, or ESKD. A lower proportion of PWH had at least 1 comorbidity than PWoH (94.1% vs 82.3%), yet their severe COVID-19 breakthrough rates remained the same as PWoH. In unadjusted analyses, moderate to severe immune suppression from HIV itself was associated with increased risk of severe COVID-19 breakthrough, as were comorbidities and a recent cancer diagnosis among PWH. Mechanical ventilation and death were rare among both PWH and PWoH with breakthrough COVID-19 and were more likely to be experienced by those older than 55 years with 1 or more comorbidity, highlighting the need for targeted risk reduction measures among older and comorbid adults.

### Limitations

This study has limitations. Our findings may not be generalizable to all PWH, as our study population had a greater proportion of men (88.9%) than found in the US population of PWH, and those with higher barriers to accessing health care (who may be at greater risk for COVID-19) were less likely to be included in our study population. Health care utilization may also differ by HIV status, which could impact inclusion with recognized breakthrough and decisions for hospitalization (outcome). Other outcome data, including mechanical ventilation and death (particularly if death occurred out of hospital) may be underascertained. All discharge diagnoses were reviewed by clinicians to increase specificity in our classification of COVID-19 hospitalization, but discharge coding can be influenced by many factors including reimbursement practices. Our findings may not be generalizable to all SARS-CoV-2 variants; however, our study was conducted while Alpha, Delta, and Omicron variants were circulating. In addition, on the basis of the timing of additional primary series doses and booster recommendations in 2021, the majority of additional doses received were timed more similarly to a booster dose than an additional dose in the primary vaccination series.

## Conclusions

Severe COVID-19 breakthrough illness was rare in our population of PWH and PWoH, with no overall differences in risk between the 2 groups; however, PWH with both moderate and severe immune suppression had an increased risk of severe COVID-19 breakthrough illness compared with PWoH. In addition to PWH with severe immune suppression, PWH with moderate immune suppression may benefit from being included in the CDC’s recommendations for those with advanced and untreated HIV. Clinicians should continue to promote risk-reduction measures among PWH. The potentially increased risk of severe COVID-19 breakthrough illness in PWH with moderate and severe immune suppression merits ongoing surveillance to inform vaccine recommendations as the pandemic persists, immunity to primary vaccine series and booster doses wane, and new variants emerge.

## Notes

Supplement. **eTable 1.** Definitions of In-Care and Criteria Used to Identify People With (PWH) and Without HIV (PWoH) **eTable 2.** Diagnosis and Procedure Codes Used to Ascertain Comorbidities and Outcomes **eTable 3.** Proportion of PWH Who Received Additional Vaccine Doses by CD4 Count at Fully Vaccinated (N=1,241) **eFigure 1.** Time From Primary Series Completion to an Additional Vaccine Dose, by HIV Status **eTable 4.** Characteristics of SARS-CoV-2 Breakthrough Infection, Both Severe and Nonsevere by HIV Status (N=3,649) **eFigure 2.** Identification of Severe COVID-19 Breakthrough Infections **eTable 5.** Severe COVID-19 Breakthrough Illness Incidence Rates and 95% Confidence Intervals, by Month and HIV Status **eFigure 3.** Histograms of the Date of Severe SARS-CoV-2 Breakthrough Infection, by HIV Status **eFigure 4.** Cumulative Incidence of Severe COVID-19 Breakthrough Illness (and 95% Confidence Intervals Represented by the Shading) Stratified by CD4 Count and HIV Status **eTable 6.** Characteristics of Participants With Breakthrough COVID-19 Illness Requiring Hospitalization, Mechanical Ventilation or in Those Who Died **eFigure 5.** Outcomes of Breakthrough Illness Among Those Hospitalized After Fully Vaccinated to Dec 31, 2021, by HIV Status (n=170 PWoH) and CD4 Count (n=23 <350 Cells/mm^3^ and n=45 ≥350 Cells/mm^3^) Among PWH
