# Supplementary material for: Analysis of Severe Illness After Postvaccination COVID-19 Breakthrough Among Adults With and Without HIV in the US
Source: JAMA Netw Open. 2022 Oct 13;5(10):e2236397. doi: 10.1001/jamanetworkopen.2022.36397 (PMC9561947; doi:10.1001/jamanetworkopen.2022.36397)
Supplement: Supplement. — eTable 1. Definitions of In-Care and Criteria Used to Identify People With (PWH) and Without HIV (PWoH) eTable 2. Diagnosis and Procedure Codes Used to Ascertain Comorbidities and Outcomes eTable 3. Proportion of PWH Who Received Additional Vaccine Doses by CD4 Count at Fully Vaccinated (N=1,241) eFigure 1. Time From Primary Series Completion to an Additional Vaccine Dose, by HIV Status eTable 4. Characteristics of SARS-CoV-2 Breakthrough Infection, Both Severe and Nonsevere by HIV Status (N=3,649) eFigure 2. Identification of Severe COVID-19 Breakthrough Infections eTable 5. Severe COVID-19 Breakthrough Illness Incidence Rates and 95% Confidence Intervals, by Month and HIV Status eFigure 3. Histograms of the Date of Severe SARS-CoV-2 Breakthrough Infection, by HIV Status eFigure 4. Cumulative Incidence of Severe COVID-19 Breakthrough Illness (and 95% Confidence Intervals Represented by the Shading) Stratified by CD4 Count and HIV Status eTable 6. Characteristics of Participants With Breakthrough COVID-19 Illness Requiring Hospitalization, Mechanical Ventilation or in Those Who Died eFigure 5. Outcomes of Breakthrough Illness Among Those Hospitalized After Fully Vaccinated to Dec 31, 2021, by HIV Status (n=170 PWoH) and CD4 Count (n=23 <350 Cells/mm3 and n=45 ≥350 Cells/mm3) Among PWH [file jamanetwopen-e2236397-s001.pdf]

Supplemental Online Content

Lang R, Humes E, Coburn SB, et al; Corona-Infectious-Virus Epidemiology Team (CIVETs) of the NA-ACCORD of IeDEA. Analysis of severe illness after postvaccination COVID-19 breakthrough among adults with and without HIV in the US. *JAMA Netw Open*. 2022;5(10):e2236397. doi:10.1001/jamanetworkopen.2022.36397

eTable 1. Definitions of In-Care and Criteria Used to Identify People With (PWH) and Without HIV (PWoH)
eTable 2. Diagnosis and Procedure Codes Used to Ascertain Comorbidities and Outcomes
eTable 3. Proportion of PWH Who Received Additional Vaccine Doses by CD4 Count at Fully Vaccinated (N=1,241)
eFigure 1. Time From Primary Series Completion to an Additional Vaccine Dose, by HIV Status
eTable 4. Characteristics of SARS-CoV-2 Breakthrough Infection, Both Severe and Nonsevere by HIV Status (N=3,649)
eFigure 2. Identification of Severe COVID-19 Breakthrough Infections
eTable 5. Severe COVID-19 Breakthrough Illness Incidence Rates and 95% Confidence Intervals, by Month and HIV Status
eFigure 3. Histograms of the Date of Severe SARS-CoV-2 Breakthrough Infection, by HIV Status
eFigure 4. Cumulative Incidence of Severe COVID-19 Breakthrough Illness (and 95% Confidence Intervals Represented by the Shading) Stratified by CD4 Count and HIV Status
eTable 6. Characteristics of Participants With Breakthrough COVID-19 Illness Requiring Hospitalization, Mechanical Ventilation or in Those Who Died
eFigure 5. Outcomes of Breakthrough Illness Among Those Hospitalized After Fully Vaccinated to Dec 31, 2021, by HIV Status (n=170 PWoH) and CD4 Count (n=23 <350 Cells/mm^3 and n=45 ≥350 Cells/mm^3) Among PWH

This supplemental material has been provided by the authors to give readers additional information about their work.

© 2022 Lang R et al. *JAMA Network Open.*

eTable 1: Definitions of In-Care and Criteria Used to Identify People With (PWH) and Without HIV (PWoH)

**Cohort**	**Description**	**"In care" or "in cohort"**	**PWH**
[a] Kaiser Permanente Mid-Atlantic States (KPMAS)	Integrated Health system	In Care – KPMAS membership for ≥1 month in 2020	HIV registry*
Kaiser Permanente Northern California (KPNC)	Integrated Health system	In Care – KPNC membership for ≥1 month between 3/1/20 and 12/31/20	HIV registry*
University of North Carolina Chapel Hill (UNC)	Medical center cohort	≥1 encounter with UNC Health in 2019 and alive as of 03/01/2020	HIV diagnosis (by ICD diagnosis code)
Veterans Aging Cohort Study (VACS)	National cohort of all PWH and 1:2 demographically-matched PWoH in care in the Veterans Health Administration (VA) system	Enrolled in VACS from 1996-2017 and alive in 2020	HIV diagnosis (presence of 1 inpatient or 2 outpatient ICD codes for HIV)

[a] The Kaiser Permanente Mid-Atlantic States and Northern California HIV Registries are databases of members diagnosed with HIV since 1998 and 1980, respectively. Primary sources used to identify HIV patients are HIV-specific laboratory tests, antiretroviral therapy, hospital-based HIV diagnosis, and diagnosis.

eTable 2: Diagnosis and Procedure Codes Used to Ascertain Comorbidities and Outcomes

Condition	ICD-10 Diagnosis Codes
**COVID-19 breakthrough infection Between Jan 1, 2020 -Dec 31, 2021**	U07.1 (specific to COVID), B34.2 (Coronavirus infection, unspecified), B97.21 (SARS-associated coronavirus causing disease classified elsewhere), B97.29 (other coronavirus as the cause of diseases classified elsewhere, J12.81 (pneumonia due to SARS-associated coronavirus, or O98.5 COVID in pregnancy, child-birth or postpartum
**Immune suppressive Conditions**	
Organ or tissue transplantation	Z94.x
Rheumatoid Arthritis	M05, M06
Lupus	M32
Cancer	C00-C14, C15-C26, C30-C39, C40-C41, C43-C44, C45-C49, C50, C51-C58, C60-C63, C64-C68, C69-C72, C73-C75, C76-C80, C81-C96, C7A, C7B, D45
Pregnancy	Z3A, O00-O16, O20-O48, O60-O77, O80-O82, O94-O9A
**Comorbidities**	
Diabetes	E11.x
End stage renal disease	N18.5 CKD stage 5, N18.6 ESRD
Hypertension	I10-I15.9, I67.4)
Obesity	E66.x, O99.21x
**Outcomes of COVID-19 Hospitalization**	
Mechanical Ventilation	**ICD-10 procedure codes:**<br>0BH17EZ: Intubation<br>5A1945Z: Respiratory Ventilation, 24-96 Consecutive Hours<br>5A1955Z: Respiratory Ventilation, Greater than 96 Consecutive Hours<br>5A1935Z: Respiratory Ventilation, Less than 24 Consecutive Hours<br>5A1522G: Extracorporeal Oxygenation, Perph VA ECMO<br>5A15A2G: Extracorporeal Oxygenation, Perph VA ECMO, Intraop<br>5A15A2H: Extracorporeal Oxygenation, Perph VV ECMO, Intraop<br><br>**CPT codes:**<br>94002: HC Ventilation Assist & Mgmt Inpatient 1st day<br>94003: HC Ventilation Assist & Mgmt Inpatient subsequent days

© 2022 Lang R et al. *JAMA Network Open.*

eTable 3: Proportion of PWH Who Received Additional Vaccine Doses by CD4 Count at Fully Vaccinated (N=1,241).

**CD4 at fully vaccinated (cells/mm^3)**	**# of COVID-19 breakthroughs in PWH**	**# who received an additional COVID-19 vaccine,** n (%)
<200	54	9 (17)
200-349	109	17 (16)
350-499	182	41 (23)
≥500	707	141 (20)
Unknown	189	34 (18)

© 2022 Lang R et al. *JAMA Network Open.*

eFigure 1: Time From Primary Series Completion to an Additional Vaccine Dose, by HIV Status

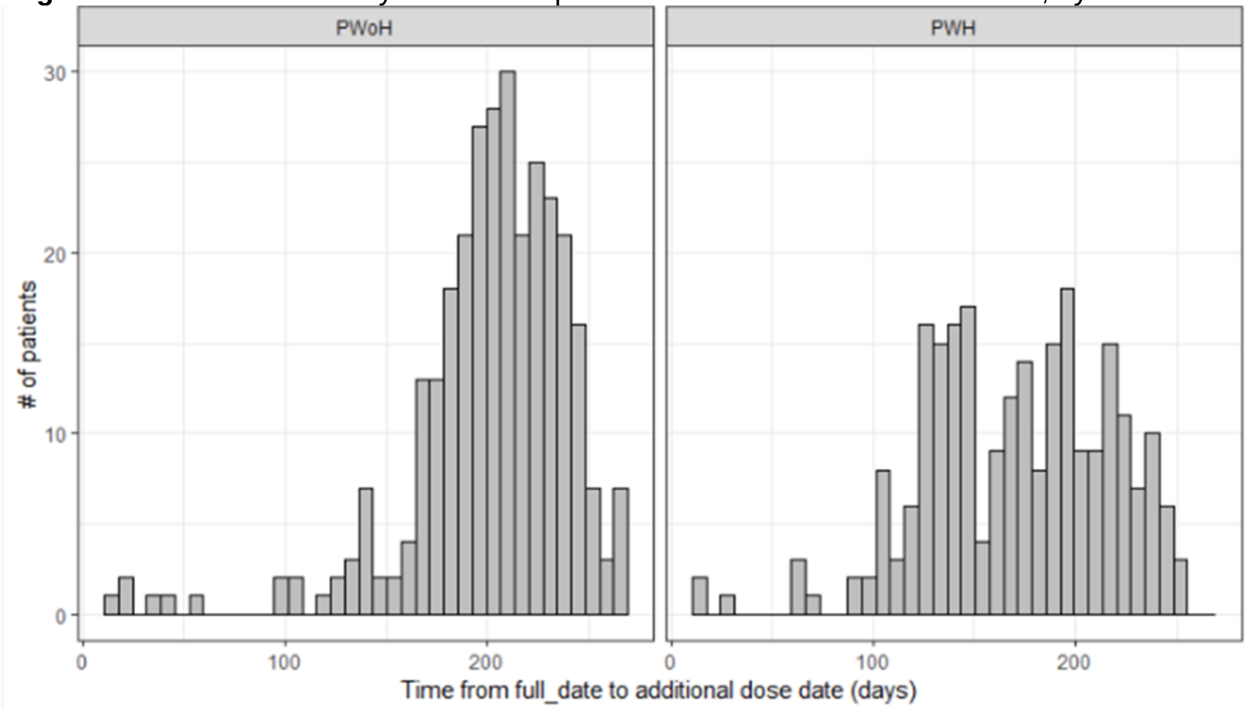

Footnotes:
PWH=People with HIV
PWoH=People without HIV

© 2022 Lang R et al. *JAMA Network Open.*

eTable 4: Characteristics of SARS-CoV-2 Breakthrough Infection, Both Severe and Nonsevere by HIV Status (N=3,649)

	Patients, No. (%)			
	PWoH N=2,408		PWH N=1,241	
**Characteristic**	**No Severe Breakthrough N = 2,238**	**Severe Breakthrough N = 170**	**No Severe Breakthrough N = 1,162**	**Severe Breakthrough N = 79**
[a] **Age (years)**				
<55	907 (40.5)	10 (5.8)	534 (46.0)	16 (20.3)
55-64	436 (19.5)	56 (32.9)	333 (28.6)	26 (32.9)
65-74	726 (32.4)	71 (41.8)	240 (20.7)	24 (30.4)
75+	506 (22.6)	33 (19.4)	55 (4.7)	13 (16.5)
**Sex**				
Male	1944 (86.9)	164 (96.5)	1,065 (91.7)	71 (89.9)
Female	294 (13.1)	6 (3.5)	97 (8.3)	8 (10.1)
**Ethnicity and Race**				
Hispanic	342 (15.3)	24 (14.1)	173 (14.9)	10 (12.7)
Non-Hispanic Black/ African American	1,060 (47.4)	91 (53.5)	515 (44.3)	40 (50.6)
Non-Hispanic White	694 (31.0)	50 (29.4)	397 (34.2)	24 (30.4)
Other	127 (5.7)	4 (2.3)	66 (5.7)	5 (6.3)
Unknown	15 (0.7)	1 (0.6)	11 (0.9)	0 (0.0)
**Month of breakthrough**				
Jan-June	231 (10.3)	16 (9.4)	129 (11.1)	5 (6.3)
July-Oct	1.024 (45.8)	108 (63.5)	585 (50.3)	54 (68.4)
Nov-Dec	983 (43.9)	46 (27.0)	448 (38.6)	20 (25.3)
**Vaccine series**				
Pfizer	1,103 (49.3)	91 (53.5)	559 (48.1)	46 (58.2)
Pfizer + 3rd dose	187 (8.4)	7 (4.1)	160 (13.8)	5 (6.3)
Moderna	629 (28.1)	47 (27.6)	285 (24.5)	16 (20.3)
Moderna + 3rd dose	90 (4.0)	5 (2.9)	67 (5.8)	1 (1.3)
J&J	215 (9.6)	20 (11.8)	82 (7.1)	11 (13.9)
J&J + 2nd dose	14 (0.6)	1 (0.6)	9 (0.8)	0 (0.0)
**COVID prior to full vaccination date**	283 (12.6)	9 (5.2)	170 (14.6)	6 (7.6)
**Comorbidities before fully vaccinated**				

© 2022 Lang R et al. *JAMA Network Open.*

Obese BMI (>=30 kg/m2)	1,132 (50.6)	86 (50.6)	402 (34.3)	32 (40.5)
Diabetes	718 (32.1)	99 (58.2)	276 (23.8)	32 (40.5)
Hypertension	1,287 (57.5)	156 (91.8)	577 (49.7)	56 (70.9)
End-stage kidney disease	41 (1.8)	22 (12.9)	34 (2.9)	4 (5.1)
Organ or tissue transplantation	27 (1.2)	16 (9.4)	17 (1.5)	1 (1.3)
Rheumatoid arthritis	38 (2.8)	6 (3.5)	13 (1.1)	1 (1.3)
Systemic lupus erythematosus	8 (0.4)	2 (1.2)	3 (0.3)	0 (0.0)
Cancer diagnosis	157 (7.0)	30 (17.6)	111 (9.5)	18 (22.8)
Pregnancy	4 (0.2)	0 (0.0)	3 (0.3)	0 (0.0)

[a] Age was categorized into a 4-level group to assess a dose response relationship among older people (i.e., 55+) that are known to be at greater risk.

© 2022 Lang R et al. *JAMA Network Open.*

eFigure 2: Identification of Severe COVID-19 Breakthrough Infections

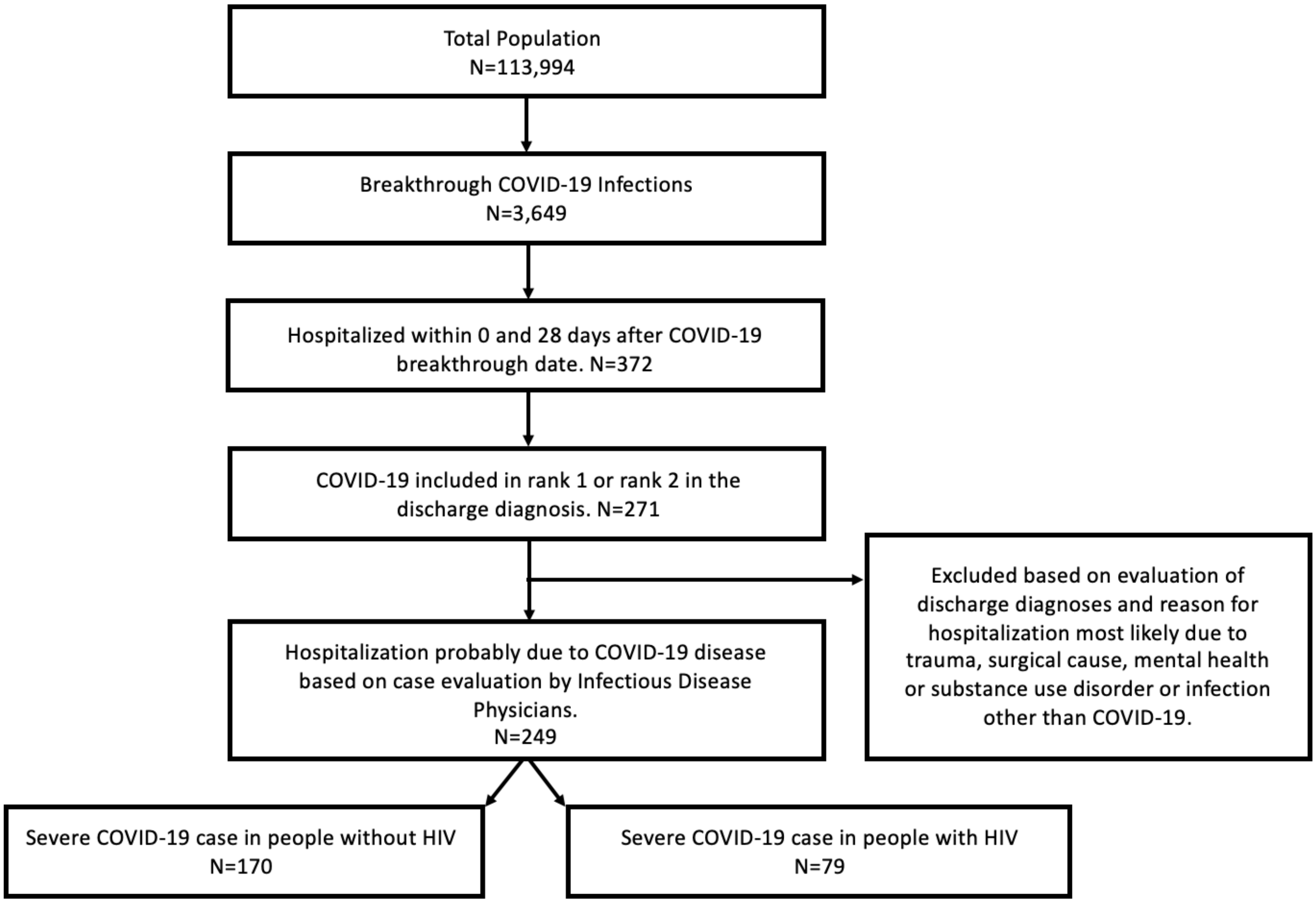

© 2022 Lang R et al. *JAMA Network Open.*

eTable 5: Severe COVID-19 Breakthrough Illness Incidence Rates and 95% Confidence Intervals, by Month and HIV Status

		# of severe COVID-19 breakthroughs	# of person-years	IR (95% CI) per 100 person years
**Total**				
	Overall	249	191.3	137.5 (117.6-159.8)
	PWH	79	67.7	116.7 (92.4-145.4)
	PWoH	170	123.6	130.2 (114.5-147.4)
**Moderna**				
	Overall	69	61.9	111.5 (86.8-141.2)
	PWH	17	21.0	81.1 (47.3-129.9)
	PWoH	52	40.9	127.1 (94.9-166.7)
**Pfizer**				
	Overall	149	110.3	135.1 (114.3-158.7)
	PWH	51	41.1	124.1 (92.4-163.2)
	PWoH	98	69.2	141.7 (115.0-172.7)
**J&J**				
	Overall	31	19.2	161.5 (109.7-229.2)
	PWH	11	5.7	194.4 (97.0-347.8)
	PWoH	20	13.5	147.8 (90.2-228.2)

**By calendar month**				
Jan	Overall	0	0.02	0.0 (0.0-19248.5)
	PWH	0	0	NA
	PWoH	0	0.02	0.0 (0.0-19248.1)
Feb	Overall	0	0.35	0.00 (0.00-1052.6)
	PWH	0	0.14	0.0 (0.0-2641.9)
	PWoH	0	0.21	0.0 (0.0-1749.8)
Mar	Overall	2	2.56	78.2 (9.5-282.5)
	PWH	0	0.90	0.0 (0.0-409.5)
	PWoH	2	1.66	120.7 (14.6-436.2)
Apr	Overall	6	6.06	99.0 (36.3-215.5)
	PWH	1	2.13	47.0 (1.2-261.6)
	PWoH	5	3.93	127.2 (41.3-296.8)
May	Overall	8	9.07	88.2 (38.1-173.8)
	PWH	3	3.13	95.8 (19.8-272.9)
	PWoH	5	5.94	84.2 (27.3-196.5)
June	Overall	3	7.33	41.0 (8.4-119.7)
	PWH	1	2.52	39.7 (1.0-221.2)
	PWoH	2	4.81	41.6 (5.0-150.3)
Jul	Overall	16	13.25	120.7 (69.0-196.1)
	PWH	4	5.60	71.4 (19.5-182.7)
	PWoH	12	7.65	156.9 (81.1-274.1)
Aug	Overall	69	44.68	154.4 (120.2-195.4)
	PWH	25	19.2	130.2 (84.3-192.2)
	PWoH	44	25.5	172.7 (125.5-231.8)
Sept	Overall	57	42.40	134.5 (101.8-174.2)
	PWH	18	13.9	129.9 (77.0-205.3)
	PWoH	39	28.5	136.7 (97.2-186.8)
Oct	Overall	20	29.25	68.4 (41.8-105.6)
	PWH	7	9.5	74.1 (29.8-152.7)
	PWoH	13	19.8	65.7 (35.0-112.3)

© 2022 Lang R et al. *JAMA Network Open.*

Nov	Overall	24	19.15	125.3 (80.3-186.5)
	PWH	7	6.0	117.3 (47.2-241.8)
	PWoH	17	13.2	128.9 (75.1-206.4)
Dec	Overall	44	36.67	120.0 (87.2-161.1)
	PWH	13	11.4	114.4 (60.9-195.7)
	PWoH	31	25.3	122.5 (83.2-173.8)

© 2022 Lang R et al. *JAMA Network Open.*

eFigure 3: Histograms of the Date of Severe SARS-CoV-2 Breakthrough Infection, by HIV Status

a) Calendar time

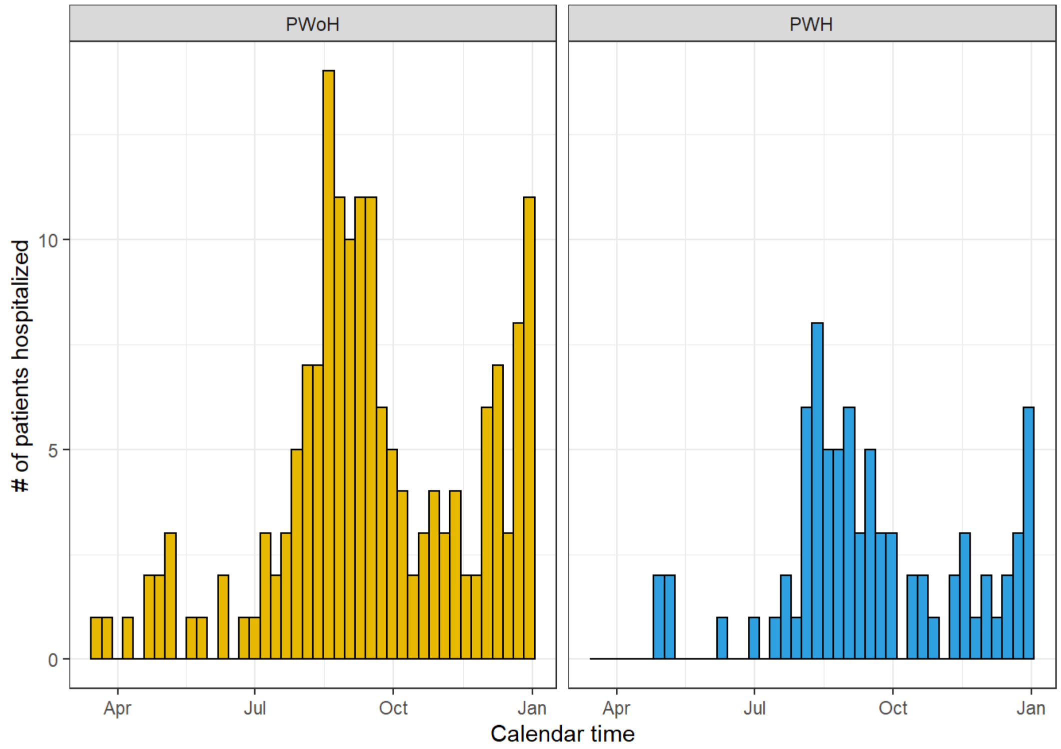

b) Time from date fully vaccinated to breakthrough

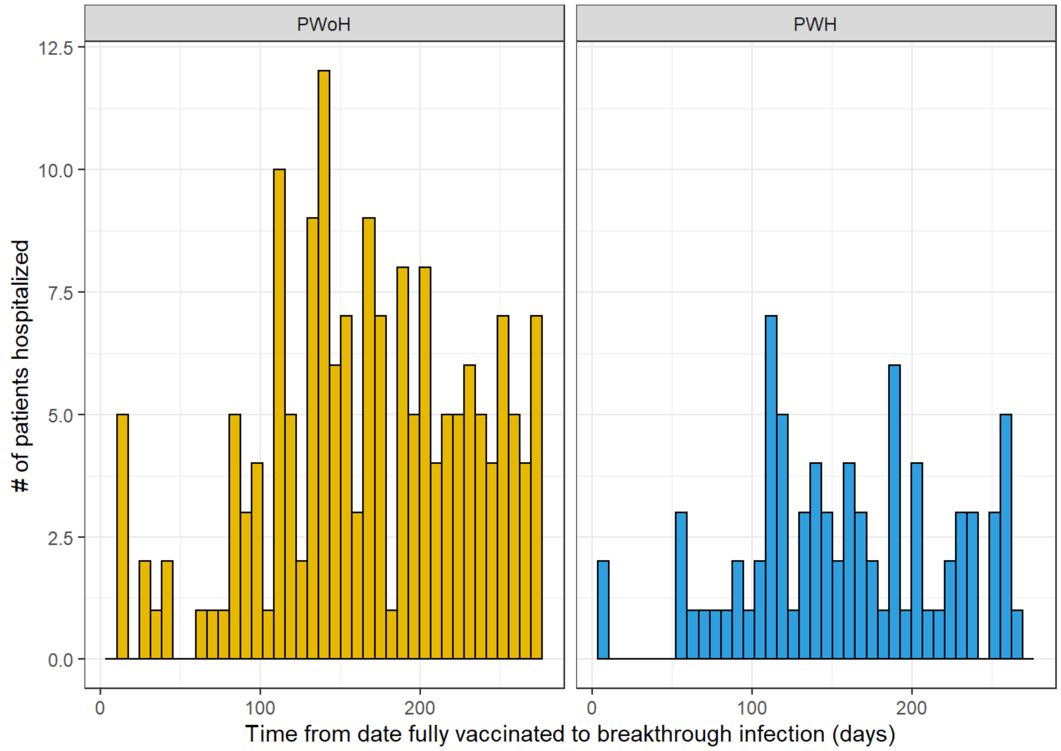

© 2022 Lang R et al. *JAMA Network Open.*

eFigure 4: Cumulative Incidence of Severe COVID-19 Breakthrough Illness (and 95% Confidence Intervals Represented by the Shading) Stratified by CD4 Count and HIV Status

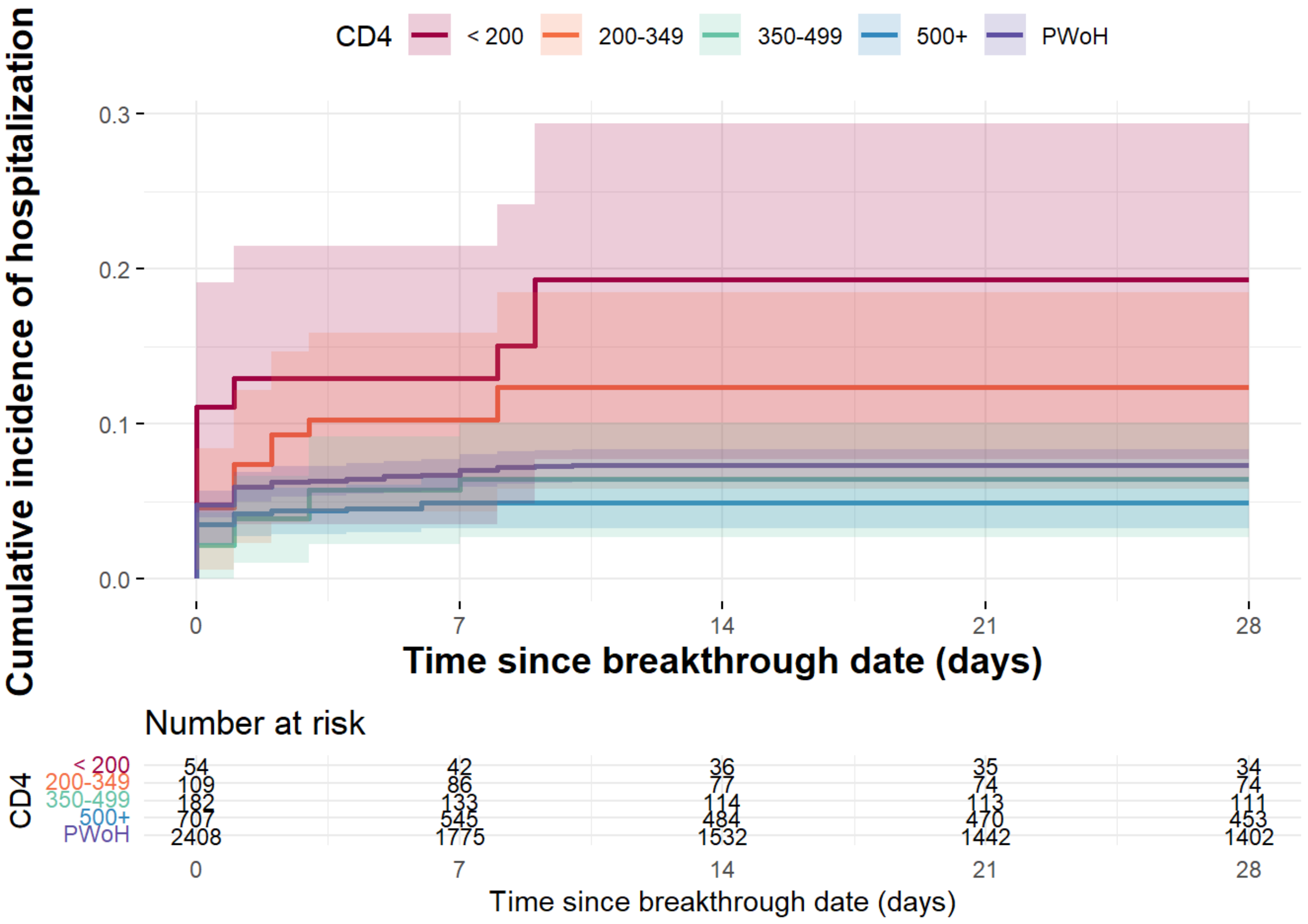

Log-rank test: p=<0.001

© 2022 Lang R et al. *JAMA Network Open.*

eTable 6: Characteristics of Participants With Breakthrough COVID-19 Illness Requiring Hospitalization, Mechanical Ventilation or in Those Who Died

	Patients, No. (%)		
**Characteristic**	Hospitalized (Severe COVID-19)	Mechanical ventilation	Death
	**N = 249**	**N = 24**	**N = 20**
**HIV Status**			
PWoH	170 (68.3)	16 (66.7)	14 (70.0)
PWH	79 (31.7)	8 (33.3)	6 (30.0)
**Age (years)**			
<55	26 (10.4)	3 (12.5)	2 (10.0)
55-64	82 (32.9)	9 (37.5)	4 (20.0)
65-74	95 (38.2)	6 (25.0)	5 (25.0)
75+	46 (18.5)	6 (25.0)	9 (45.0)
**Sex**			
Male	235 (94.4)	20 (83.3)	18 (90.0)
Female	14 (5.6)	4 (16.7)	2 (10.0)
**Ethnicity and Race**			
Hispanic	34 (13.7)	4 (16.7)	4 (20.0)
Non-Hispanic Black/African American	131 (52.6)	18 (75.0)	11 (55.0)
Non-Hispanic White	74 (29.7)	2 (8.3)	5 (25.0)
Other	9 (3.6)	0 (0.0)	0 (0.0)
Unknown	1 (0.4)	0 (0.0)	0 (0.0)
**Month of breakthrough**			
Jan-June	21 (8.4)	3 (12.5)	3 (15.0)
July-Oct	162 (65.1)	16 (66.7)	13 (65.0)
Nov-Dec	66 (26.5)	5 (20.8)	4 (20.0)
**COVID prior to vaccination**	15 (6.0)	0 (0.0)	0 (0.0)
**Vaccine Series**			
Primary Series Only	230 (92.4)	21 (87.5)	18 (90.0)
Primary Series + additional dose	19 (7.6)	3 (12.5)	2 (10.0)
**Obese BMI (>=30 kg/m^2)**	118 (50.0)	20 (87.0)	13 (65.0)
Unknown	13 (5.2)	1 (4.2)	1 (5.0)
**Diabetes**	131 (52.6)	15 (62.5)	13 (65.0)
**Hypertension**	212 (85.1)	23 (95.8)	19 (95.0)
**End-stage kidney disease**	26 (10.4)	5 (20.8)	6 (30.0)
**Organ or tissue transplantation**	17 (6.8)	5 (20.8)	4 (20.0)
**Rheumatoid arthritis**	7 (2.8)	3 (12.5)	2 (10.0)
**Systemic lupus erythematosus**	2 (0.8)	0 (0.0)	0 (0.0)
**Cancer diagnosis**	48 (19.3)	5 (20.8)	5 (25.0)
**CD4 at ART initiation, median (IQR)**	399 (212,546)	376 (324,417)	398 (354,855)
Unknown	210 (84.3)	20 (83.3)	1 (16.7)
**AIDS before fully vaccinated**	29 (36.7)	3 (37.5)	3 (50.0)
Unknown	170 (68.3)	16 (66.7)	0 (0.0)

© 2022 Lang R et al. *JAMA Network Open.*

**CD4 at fully vaccinated, median (IQR)**	498 (305,746)	352 (172,542)	352 (291,423)
Unknown	181 (72.7)	18 (75.0)	0 (0.0)
**Supressed HIV RNA at fully vaccinated**	59 (86.8)	5 (83.3)	4 (100.0)
Unknown	181 (72.7)	18 (75.0)	0 (0.0)

eFigure 5: Outcomes of Breakthrough Illness Among Those Hospitalized After Fully Vaccinated to Dec 31, 2021, by HIV Status (n=170 PWoH) and CD4 Count (n=23 <350 Cells/mm^3 and n=45 ≥350 Cells/mm^3) Among PWH

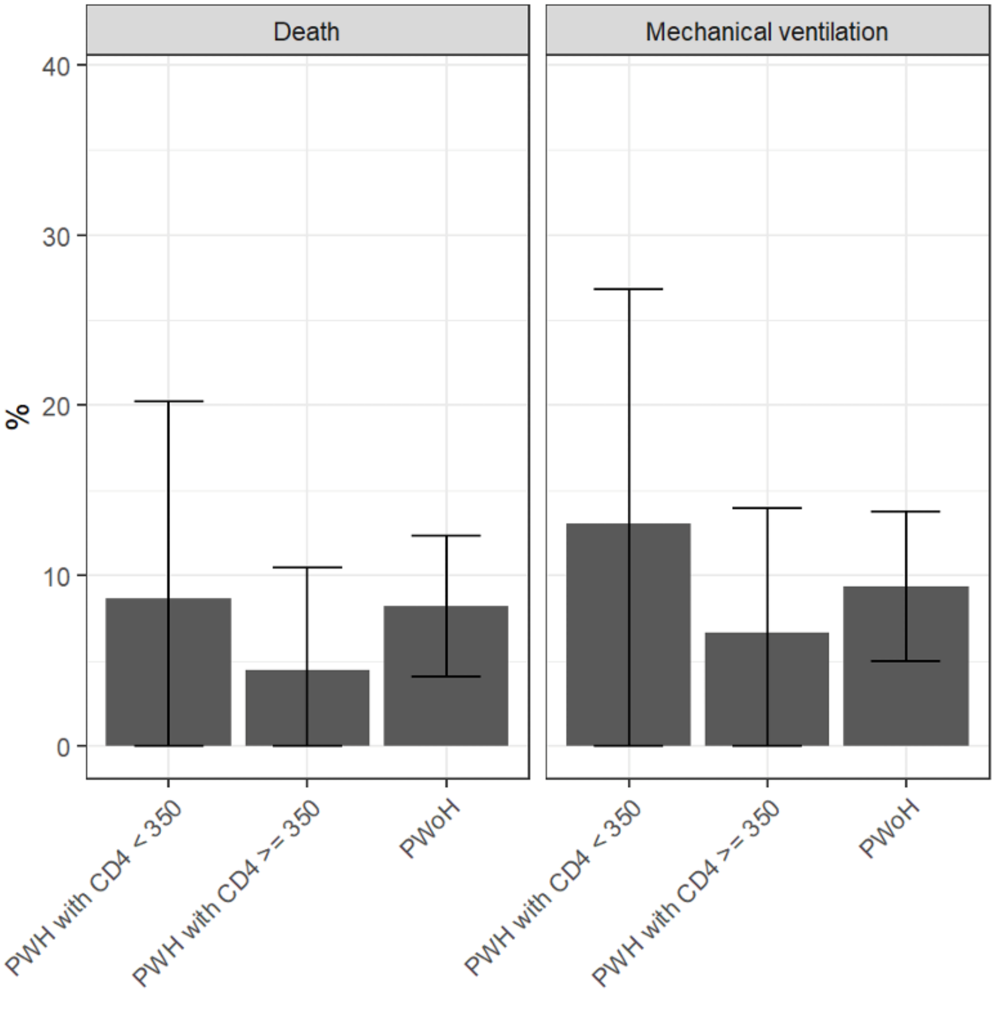

© 2022 Lang R et al. *JAMA Network Open.*
